# In Patients not Suitable for Generalised Anaesthesia, Surgery for Necrotising Fasciitis under Spinal Anaesthesia should be Considered

**DOI:** 10.5704/MOJ.2503.019

**Published:** 2025-03

**Authors:** J Finsterer, S Zarrouk

**Affiliations:** 1 Neurological Department, Neurology and Neurophysiology Center, Vienna, Austria; 2 Department of Genomic Platform, University of Tunis El Manar, Tunis, Tunisia

Dear editor,

We read with interest the article by Faris *et al*^1^ about a 54-year-old man who suffered from necrotizing fasciitis secondary to enterococcal infection but, due to severe heart failure and anticoagulation with dabigatran^1^, did not undergo resection of the necrosis, which is the gold standard in the treatment of fasciitis^2^, but instead underwent conservative therapy. Despite this strategy, the patient partially recovered at the three-month follow-up with partial detachment of the eschar with underlying healthy granulation tissue and was able to use a wheelchair^1^. Some ambiguities should be clarified.

First, we do not agree with the assumption that the fasciitis was caused by dabigatran, as stated in the title of the study^1^. So far, there is no evidence that dabigatran itself can directly cause fasciitis. Necrotizing fasciitis is a rare but serious bacterial infection. It can very quickly develop into a life-threatening emergency^3^. Early symptoms include fever, severe pain and a rapidly spreading infection. People with necrotizing fasciitis require immediate hospital treatment, antibiotics and surgery^3^.

The second point is that it was not stated how the fasciitis was diagnosed. Was a biopsy or MRI performed on admission? Was the fasciitis diagnosed simply on the basis of the clinical picture on admission, as shown in Figure 1?

The third point is that the underlying pathophysiology of the fasciitis was not clarified. By what route did the patient become infected with enterococci? Did the patient have a history of cuts and scratches, burns and scalds, insect bites, previous surgery or medication injected into the skin? Has he had skin bleeding from the anticoagulant?

The fourth point is that it is not understandable why the surgery for fasciitis was not performed under spinal anaesthesia^4^. It is also incomprehensible why the anticoagulant effect of dabigatran was not antagonised with Idarucizumab before the operation.

The fifth point is that the indication for dabigatran is unclear. Was the patient on anticoagulation for severe heart failure, atrial fibrillation, deep vein thrombosis or pulmonary embolism? There was also no mention of whether the coagulation parameters on admission indicated that the patient was actually anticoagulated on admission. Was the aPTT prolonged? Was the serum level of dabigatran measured? It is also unclear what the authors mean by the term “coagulopathy”. Was the patient over- or under-coagulated? Did he also have an inherited or acquired coagulopathy?

The sixth point is that the cause of the severe heart failure was not specified^1^. Was it hypertrophic or dilated cardiomyopathy, myocarditis, coronary artery disease or Takotsubo syndrome? Did the patient have classic cardiovascular risk factors such as hypertension, diabetes, smoking, hyperlipidemia or atrial fibrillation? What was the current medication at the time of admission?

The seventh point is that the reason for the resuscitation was not stated^1^. Did the patient require resuscitation for asystole, ventricular fibrillation, heart failure, pulmonary embolism or septic shock? The explanation for CRP is crucial for the interpretation of the course of the disease and the underlying pathophysiology.

In summary, it can be said that this interesting study has limitations that relativise the results and their interpretation. Removing these limitations could strengthen the conclusions and reinforce the message of the study. All unanswered questions need to be clarified before readers can uncritically accept the study's conclusions. In patients with necrotizing fasciitis of the legs, severe heart failure and anticoagulation with dabigatran, debridement under spinal anaesthesia after antagonisation with idarucizumab should be considered.
