# Supplementary material for: In Patients not Suitable for Generalised Anaesthesia, Surgery for Necrotising Fasciitis under Spinal Anaesthesia should be Considered
Source: Malays Orthop J. 2025 Mar;19(1):133–5. doi: 10.5704/MOJ.2503.019 (PMC12022705; doi:10.5704/MOJ.2503.019)
Supplement: Related texts

# Authors’ Reply to the Letters to the Editor

**Published:** 2025-03

**Authors:** Ahmad Faris Mohd Khairuddin, Muhamad Izzuddin Mohd Sharif, Mohamad Norazrin Mohd Abas, Thiru Kumaran Mootha Kumaran

**Affiliations:** Department of Orthopaedic Surgery, Miri Hospital, Miri, Sarawak

We value the editorial board’s detailed review of our case report and are pleased to address the concerns highlighted. We agree there is no direct evidence linking dabigatran to necrotizing fasciitis (NF). Our report aimed to highlight anticoagulation's potential role in exacerbating soft tissue haemorrhage, contributing to NF1. A more precise title, such as "Dabigatran-Associated Enterococcus Necrotizing Fasciitis in a Medically Unfit Patient," could help avoid potential misinterpretations.

NF was diagnosed clinically based on the rapid disease progression, including blackish discoloration, haemorrhagic bullae, and systemic sepsis^1,2^. The LRINEC score of 8, though not mentioned initially, strongly supported the diagnosis. While magnetic resonance imaging (MRI) is a valuable diagnostic tool in well-equipped centres, its utility is most effective in cases with atypical or mild presentations. MRI or biopsy was not pursued to avoid delays, as the presentation was unequivocal.

The infection route remains uncertain. The absence of trauma or wounds, coupled with anticoagulation-induced hematomas, likely provided a nidus for bacterial growth. Enterococcus sp., a gut commensal, may have originated from gastrointestinal translocation or hematogenous spread2.

Given the patient’s critical condition, severe heart failure (ejection fraction of 7%), coagulopathy, and multiorgan dysfunction, surgery under spinal anaesthesia, even with Idarucizumab reversal, posed substantial risks. Additionally, Idarucizumab was not readily available. A multidisciplinary team concluded, with patient and family agreement, that conservative management was the safest course.

Dabigatran was prescribed for left ventricular mural thrombus prophylaxis following an echocardiogram identifying severe heart failure with global hypokinesia. Admission findings of prolonged aPTT and elevated INR indicated excessive anticoagulation, exacerbated by the sepsis-related coagulopathy.

The patient had dilated cardiomyopathy due to long-standing hypertension, accompanied by hyperlipidaemia and smoking, with a hallmark severely reduced ejection fraction. He was on beta-blockers, ACE inhibitors, and dabigatran at admission.

The patient initially required resuscitation due to septic shock, which was secondary to the NF. Elevated CRP levels further supported the diagnosis of systemic inflammation and sepsis which also helped in monitoring the progression of the disease and the patient's recovery during treatment^3^.

In conclusion, we recognise the study's limitations and appreciate the suggestions to strengthen its conclusions. However, we maintain that conservative management was appropriate given the patient’s high perioperative risk. We trust this explanation provides further clarity on our approach and the complexities involved in this case.

## References

[1] Wallace HA, Perera TB (2023). Necrotizing Fasciitis..

[2] Faris MK, Norazrin MA, Izzuddin MS, Thiru MK (2024). Dabigatran-induced Enterococcus Necrotizing Fasciitis in Medically Unfit Patient with Successful Conservative Treatment Outcome: A Case Report.. Malays Orthop J..

[3] Anush MM, Ashok VK, Sarma RI, Pillai SK (2019). Role of C-reactive Protein as an Indicator for Determining the Outcome of Sepsis.. Indian J Crit Care Med..
